# Medical students’ attitudes towards communication skills training: a longitudinal study with one cohort

**DOI:** 10.3205/zma001503

**Published:** 2021-09-15

**Authors:** Roger Ruiz-Moral, Diana Monge Martin, Cristina Garcia de Leonardo, Sophia Denizon, Alvaro Cerro Pérez, Fernando Caballero Martínez

**Affiliations:** 1 Universidad Francisco de Vitoria, Facultad de Ciencias de la Salud, Escuela de Medicina, Departamento de Educación Médica, Madrid, Spain; 2 Instituto Maimónides de Investigación Biomédica de Córdoba (IMIBIC), Córdoba, Spain; 3 Universidad Francisco de Vitoria, Facultad de Ciencias de la Salud, Medicina Familiar y Preventiva, Madrid, Spain; 4 Universidad Francisco de Vitoria, Facultad de Ciencias de la Salud, Neurofisiólogo, Madrid, Spain; 5 Universidad Francisco de Vitoria, Facultad de Ciencias de la Salud, Escuela de Medicina, Madrid, Spain; 6 Universidad Francisco de Vitoria, Escuela de Ciencias de la Salud, Madrid, Spain

**Keywords:** communication skills, experiential learning, longitudinal study, medical education, medical students

## Abstract

**Objectives: **To explore medical students’ attitudes towards communication skills and the evolution of these attitudes from their first to fourth academic years.

**Methods:** A cohort of 91 medical students completed the Communication Skills Attitudes Scale (CSAS) at the beginning of their medical studies and at the end of their fourth year after having engaged in a training program in communication skills with experiential characteristics (individual encounters with simulated patients, observations in small groups, feedback, and practice). We analyzed students’ positive and negative global attitudes and their affective, cognitive, and respect dimensions towards learning communication skills.

**Results:** Medical students’ attitudes toward communication skills declined from their first (52.8) to fourth year (49.6) (p=.011). Along with this significant decrease in positive attitudes, a significant increase in negative attitudes toward communication skills was also observed in trained students (32.2 vs. 34.2; p=.023). The decline in students’ attitudes mainly involves a decline in their affective (51.4 vs. 47.3, p=.001) but not cognitive (18.3) attitudes. Female students have more positive attitudes towards communication skills than male students.

**Conclusions: **The decline in students’ attitudes, mainly in the affective dimension, could be related to their accumulated learning experiences during the learning process and particularly their experiential training in communication skills. Nevertheless, the importance students give to communication skills in the cognitive dimension remains unchanged. Students’ gender also seems to influence their attitudes. Further research is needed to assess the role of other factors involved in this decrease in positive and affective attitudes.

## Zusammenfassung

**Ziele:** Erforschung der Einstellung von Medizinstudent*innen gegenüber Kommunikationsfähigkeiten und die Entwicklung dieser Einstellung zwischen dem ersten und vierten akademischen Jahr.

**Methoden: **Eine Kohorte von 91 Medizinstudent*innen beteiligte sich zu Beginn des Medizinstudiums und zum Ende des vierten Jahres am Communication Skills Attitudes Scale (CSAS)-Test; Letzteres nachdem sie an einem Trainingsprogramm zu erfahrungsbasierten Kommunikationsfähigkeiten teilgenommen hatten (individuelle Begegnungen mit simulierten Patienten, Beobachtungen in Kleingruppen, Feedback und Praxis). Wir analysierten die globalen positiven und negativen Einstellungen der Studierenden sowie deren affektive, kognitive und respekt-bezogene Dimensionen gegenüber dem Erlernen von Kommunikationsfähigkeiten.

**Ergebnisse:** Die Einstellung von Medizinstudent*innen gegenüber Kommunikations-fähigkeiten sank von ihrem ersten (52,8) zum vierten Jahr (49,6) (p=.011). Zusammen mit dieser signifikanten Abnahme einer positiven Einstellung, beobachteten wir einen signifikanten Anstieg einer negativen Einstellung gegenüber Kommunikationsfähigkeiten bei den ausgebildeten Studierenden (32,2 gegenüber 34,2; p=.023). Das Absinken der Einstellung der Studierenden umfasst hauptsächlich eine Abnahme ihrer affektiven (51,4 gegenüber 47,3, p=.001), jedoch nicht ihrer kognitiven (18,3) Einstellung. Weibliche Studierende haben eine positivere Einstellung gegenüber Kommunikationsfähigkeiten als männliche Studierende.

**Schlussfolgerungen: **Das Absinken der Einstellung der Studierenden, hauptsächlich deren affektiver Aspekt, könnte zusammenhängen mit ihren akkumulierten Lernerfahrungen während des Lernprozesses und insbesondere mit ihrem erfahrungsbasierten Training der Kommunikationsfähigkeiten. Trotzdem bleibt die Bedeutung, die Studierende Kommunikationsfähigkeiten in deren kognitiver Dimension beimessen, unverändert hoch. Das Geschlecht der Studierenden scheint ebenfalls einen Einfluss auf ihre Einstellung zu haben. Weitere Forschungen sind nötig, um die Rolle anderer Faktoren zu untersuchen, die an dieser Abnahme positiver und affektiver Einstellungen beteiligt sind.

## 1. Introduction

The ways in which communication skills (CS) are taught to medical students, including assessments of such skills and students’ experiences of different educational methods, along with other socio-demographic features, can influence students’ attitudes towards this type of learning and thus influence the effectiveness of CS learning programs [1], [2], [3], [4], [5]. While some authors have pointed out differences in students’ attitudes towards didactic and experiential methodologies [6], preferring the latter, others [7] assert the opposite and attribute an increase in negative attitudes to the emotional distress caused by training through direct or video observation. The same can be said of interactions with real or simulated patients and the subsequent feedback on students’ performance, either individually or in small groups [8], [9], [10]. This is important because attitudes are strong behavioral indicators [11], [12], [13] and students’ attitudes towards communication skills may influence the amount of time they dedicate to learning such skills [14] and how they will put them into practice when treating patients [15]. Several studies have described how students’ attitudes towards CS decline the further they progress with their medical studies [16], [17], [18], [19], [20]. In such processes, medical students start their studies with an idealistic and altruistic attitude and then finish feeling cynical and detached [21]. 

In a preliminary study [22], we explored attitudes toward CS learning in two independent groups of medical students, one at the beginning of their medical studies and with no learning experience in CS (first-year medical students) and the other (fourth-year medical students) after having received experiential training at our medical school. The results of this study show that students who had been given training in communication skills had fewer positive attitudes toward CS, particularly affective attitudes, than first-year untrained students, suggesting that students’ attitudes towards communication skills may decline as a result of communication skills training. In order to test this hypothesis, we designed this study to explore how students’ attitudes evolve over the course of their medical training. To this end, we longitudinally followed a cohort of medical students from their first to fourth years at our medical school, where students engage in two early clinical immersion periods and later receive two years of training experience in communication that features both experiential and interactive characteristics. 

## 2. Methods

### 2.1. Study design, participants, and setting

This research involved a longitudinal prospective study with a cohort of medical students. All medical students in their first year were invited to participate in this study. After obtaining their prior consent, students completed a CSAS questionnaire anonymously in a seminar at the beginning of their first year and again when they finished their fourth year. This questionnaire took no longer than 20 minutes to complete. The completed questionnaires were collected anonymously and sent for analysis. 

#### 2.2. Structure and teaching activities in communication training 

**Figure 1:**
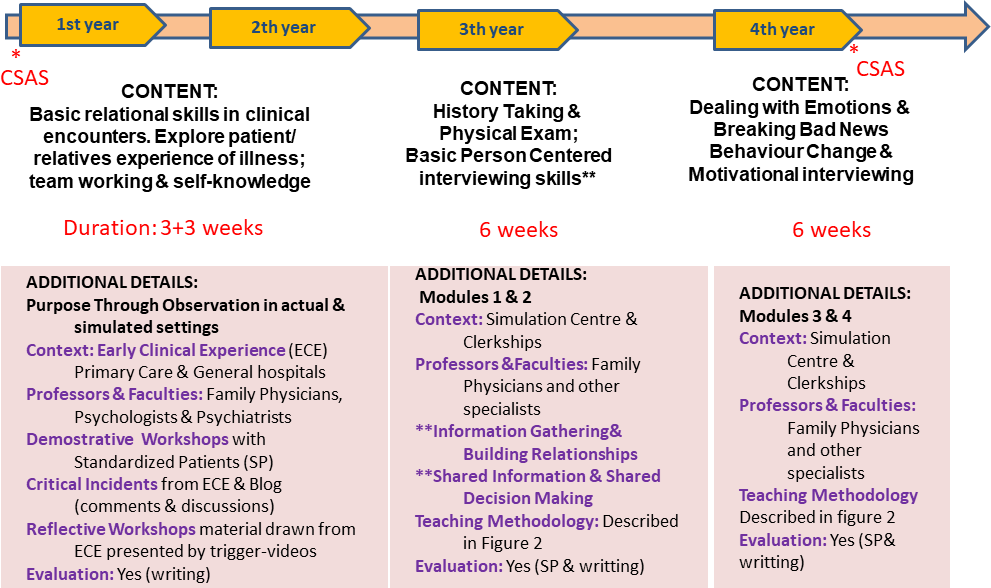
Overview on communication training curriculm

Medical students at our Faculty of Medicine are required to engage in patient-physician communication training (see figure 1 (Fig. 1)). In their first and second years, students go through early clinical immersion periods in primary care and mental health services where they develop observational tasks on communication behaviors that are later discussed in workshops. Later in their third and fourth years and for six weeks each year, students work in depth with patients in consultations in hospital and primary care. In their third year, they receive basic training focused on communication skills for performing “person-centered interviews.” The fourth year is devoted to more specific and advanced communication skills. The overall training program has four modules. The objective of the first module is to train students in the use of communication skills to obtain relevant clinical information and to establish suitable doctor-patient relationships. The objectives of the second module focus on providing information and sharing information during the decision-making process. The aims of the third module relate to dealing with emotions in consultations and giving patients bad news. The final module introduces students to communicative strategies to influence patients’ behavior, mainly through motivational interviewing. The first two modules are taught during the third year and the final two in the fourth year. All course modules involve the following activities. 

##### 1. Demonstrative and small-group work sessions

Addressing specific interview topics and communication skills. Students work in small groups on situations depicted in videos and clinical cases. These sessions involve individual reflection and plenaries with a discussion and presentation of evidence and analysis of the strategies proposed. 

##### 2. Workshops with simulated patients

Some students interview a simulated patient (SP) while the rest observe and evaluate the interaction in terms of objectives achieved and skills used. After each encounter, the student receives feedback from their peers, the SP, and teaching staff (faculty). 

##### 3. Group practice and reports

Additional groups of four students are organized to interview, observe, and provide feedback to each other. In these encounters, the students engage in roleplay. Points of interest are collected in a notebook for each student with information about the development of their skills and the experience in general. 

##### 4. Interviews with SP

**Figure 2:**
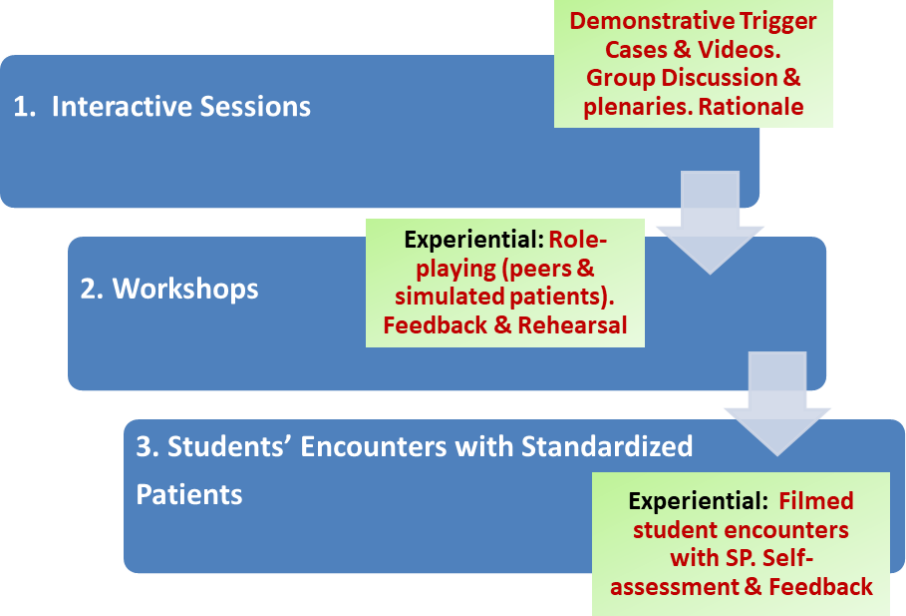
General characteristics of communication skills program.

All students engage in two or three videotaped encounters with SPs in every module. This is done at the Simulation Center, which is equipped with a built-in video recording system that allows videos to be viewed online for assessment. After each interview, all students complete a quantitative self-assessment form (1 Deficient, 5 Excellent) of their interview skills and have the chance to make comments. Subsequently, each student receives individualized feedback from the teaching staff using the same qualitative-quantitative methodology. Figure 2 (Fig. 2) shows the general teaching program for all modules of this program.

#### 2.3. Communication Skills and Attitudes Scale (CSAS) 

The Communication Skills and Attitudes Scale (CSAS) is a 26-item scale (ranging from 1, strongly disagree, to 5, strongly agree) developed to explore medical students’ attitudes towards CS education [23]. The items evaluate students’ perceptions of the ways in which CS is taught, the importance of developing CS to pass exams and be a good doctor, and the use of CS to show respect to patients and colleagues. We used a validated Spanish version based on the original English version [22]. The initial psychometric analysis of the scale performed by Rees et al. [23] identified two sub-scales or factors, each with 13 items and representing positive and negative attitudes towards communication skills learning. In this previous study [23] with 490 students, both sub-scales showed a satisfactory reliability and internal consistency. In a subsequent study conducted with 1,833 students at four Norwegian faculties of medicine, Anvik et al. [24] identified three factors in the CSAS that differed from those previously described by Rees et al.: factor 1, “learning,” mainly measures students’ feelings about how communication skills are taught and mainly reflects the affective aspects of their attitudes. factor 2, “importance,” encompasses students’ attitudes towards CS, mainly reflecting their basic cognitive attitudes and values. Finally, factor 3 is “respect,” as all the items establish that communication skills are useful for students when it comes to respecting patients and colleagues. In our data analysis process, we considered Rees et al.’s factors as well as those identified by Anvik et al.: positive and negative attitudes and affective, cognitive, and respect attitudes. 

#### 2.4. Data analysis

An exploratory analysis of the data was first performed to establish the distribution of the continuous variables. The comparison student t-test means and the Pearson correlation coefficients were used. Finally, to assess possible associations between different variables and the CSAS global and affective dimensions score, a multivariate analysis was performed (linear regression).

The study protocol was approved by the UFV School of Health Sciences’ Research and Ethics Committee (PRPI_Medicina_UFV_3/2016).

## 3. Results

The questionnaire was answered by 114 students in their first year and 91 students in their fourth year (79.8%) (average age: 18.7 and 22 years in the first and fourth years, respectively). Sixteen students dropped out of the university, generally for non-academic reasons (they went on to other universities); five students were not yet enrolled in the last module in year 4 and two did not fill out the survey. No data was obtained on these students. Most of the students were female, with 87 (76.3%) female students responding in their first year and 71 (76,3%) in their fourth year; 30 students (38%) had a parent who was a doctor; and 74 students (73%) had completed their sixth-form studies (baccalaureate) at private schools (mostly religious schools). 

**Figure 3:**
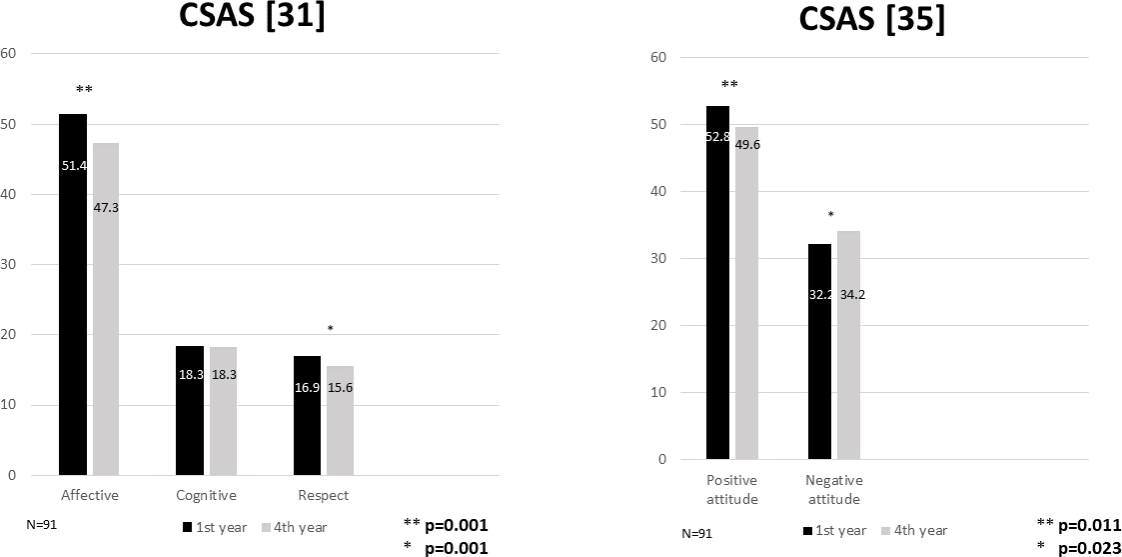
Medical students' scores in the CSAS [31], [35] subscales in their first and fourth years

The results of the CSAS for the global attitude and its three dimensions (affective, cognitive, and respect) in the first and fourth years for both genders are shown in figure 3 (Fig. 3) and table 1 (Tab. 1), respectively. In the multivariate linear regression model that took the global positive attitude score on the CSAS as a dependent variable, significant positive associations were found with students’ self-perception of their communication skills (β=4.013; p=0.000) and being female (β=2.692; p=0.047), and negative associations were identified with the upper course (fourth-year) (β=1.154; p=0.003). Being male (β=2.692; p=0.047) and in the upper course (fourth-year) (β=1.154; p=0.003) were significantly associated with global negative attitude scores in the model that took this variable as a dependent variable. The students’ self-assessment of their own CS had a positive correlation with a generally positive attitude in their first and fourth years (0.300, p: 0.002 and 0.25, p: 0.021) and with the affective domain in their first year (0.32, p: 0.001); a negative correlation with a negative attitude was found in first-year students (0.270, p: 0.04). All the rest of the correlations in both academic years were non-significant.

## 4. Discussion

Our medical students’ attitudes toward CS declined from their first to fourth year. Along with this significant decrease in positive attitude, a significant increase in negative attitude toward CS learning was also observed in these trained students. The results observed in this cohort of students are similar to those we observed in a previous study with two independent groups of students: one in their first year and one in the fourth year [22]. Likewise, this decline in medical students’ attitudes is supported by the results obtained by other researchers [6], [16], [18], [19], [25]. In a more recent study using the same measurement instrument performed with two cohorts of medical students that differed only by having received CS training or not, students’ attitudes towards CS teaching during clerkship and attitudes focused on patients worsened in students trained in CS [26]. 

Analyzing these results with the CSAS sub-scales proposed by Anvik et al., our findings are also consistent with those obtained by other authors [7], as they indicate that the decline in medical students’ attitudes mainly involves a decline in their affective attitudes. This reflects their feelings and experiences regarding the way in which CS is taught rather than the importance students give to CS in their studies and clinical practice (cognitive dimension), which remains unchanged. According to these studies, experiential training is pivotal for success in teaching and learning communication skills [27], [28], [29]. However, both our study and those by Bombeke et al. [26] and in particular by Anvik et al. [7] move towards the hypothesis that this type of teaching may be producing significant changes in students’ attitudes towards the teaching received rather than the importance they give to CS. After analyzing these results, we conducted a qualitative study to clarify our own students’ points of view and experiences regarding the CS training they received in previous years [30]. This study revealed a variety of associated topics; although most students viewed communication topics as useful and practical, they confessed that they had encountered problems in the small groups in which they were required to interview an SP in front of their peers, mainly due to difficulties in putting theory into practice and feelings of embarrassment. The summative assessment of these CS also brought about a wide range of negative feelings and was identified as the main source of stress. It seems that this method of teaching CS often makes students feel uncomfortable and anxious [10], [31] and leads to the development of negative affective attitudes. In this same study, our students pointed out that receiving detailed and constructive feedback for learning new skills in a careful and thoughtful manner was a very gratifying and productive experience. However, although this may mitigate the stress they experienced [10], [32], it does not seem to be sufficient to make the feelings of discomfort and embarrassment disappear, nor does it address the distress of holding the first interviews, the compulsory nature of this learning, or performing a summative assessment [33] based on the interviews with simulated patients. Finally, similar to the findings of other studies [6], [34], [35], we found that female students seemed to have more positive attitudes towards CS than male students. These results indicate that the negative influence of factors related to the implementation of experiential methodologies may be influenced by gender as well as other factors unexplored in this study such as workload, stress, or real scenarios with patients. 

### Limitations

This study has some limitations that make it difficult to generalize the results: the study was carried out at a single university with students belonging to the same academic cohort. There are also obvious elements of uncertainty regarding the validity of the CSAS and the use of statistical procedures. The CSAS was not designed to differentiate between cognitive and affective attitudes. Nevertheless, the fact that other authors applied it to a large sample of students with congruent results [7] supports its validity. We pointed out the existence of other variables that were not examined in this study that may influence these results, which may be related to student's new responsibilities or decreases in students’ empathy [18], [36], [37].

## 5. Conclusions

The findings of this study support the hypothesis that students’ attitudes towards CS decline as a result of CS training. However, students’ attitudes towards the cognitive dimension remain unchanged. Students’ gender and their accumulated learning experiences during the learning process seem to influence their attitudes. Learning CS with experiential methods seems to be challenging for students on a personal level. Educators should keep this in mind when designing CS lessons and emphasize personalization as much as possible. Further research is needed to assess the role of other factors involved in this decrease of positive attitudes toward CS.

## Competing interests

The authors declare that they have no competing interests. 

## Figures and Tables

**Table 1:**
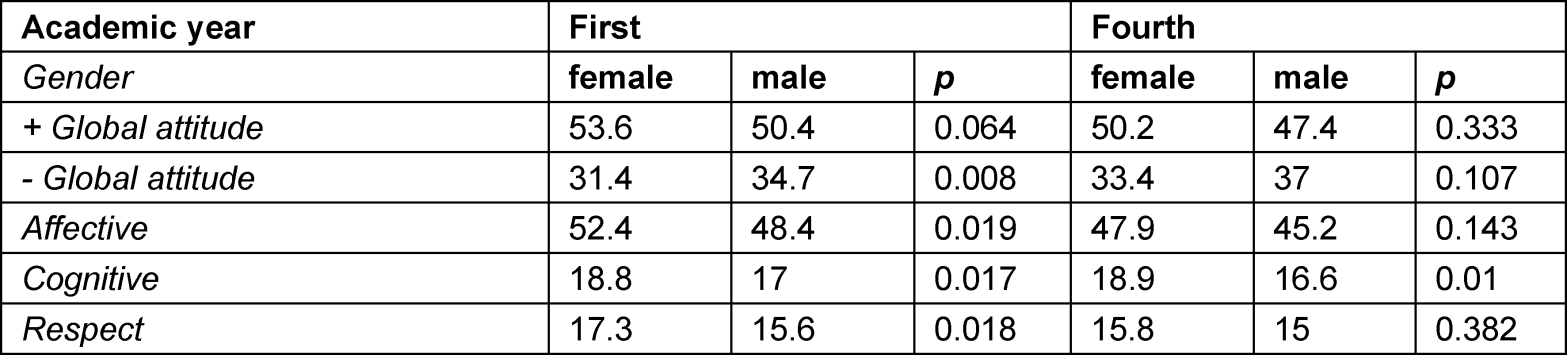
CSAS scores for global attitude and its three dimensions (affective, cognitive, and respect) in first- and fourth-year students of both genders
